# Envisioning supportive and safe learning environments: A dialogical study with LGBTQ+ adolescents living with chronic conditions or diagnoses: Lourdes Cantarero Arevalo

**DOI:** 10.1093/eurpub/ckac129.678

**Published:** 2022-10-25

**Authors:** L Cantarero Arevalo, A Skov Nielsen, L Slyngbord

**Affiliations:** 1 Faculty of Health and Medical Sciences, University of Copenhagen, Copenhagen, Denmark; 2 Faculty of Humanities, University of Copenhagen, Copenhagen, Denmark

## Abstract

**Background:**

LGBTQ+ adolescents living with mental conditions are affected by stigma based on their health status, sexual orientation, and/or gender identity/expression (SOGIE), especially when navigating their learning environments. Our aim is to gain detailed insights into how LGBTQ+ adolescents living with mental conditions vision their learning environments so that they can feel safe and supported enough to freely disclose their SOGIE and health status, and hence thrive.

**Methods:**

Aware of the participants’ vulnerabilities, a dialogical narrative-based approach was used to gather thick descriptions and deep insight, while applying the “seven C's”: conversation, curiosity, context, complexity, challenge, caution, and care (Frank, 2019). Recruitment was done through LGBTQ+ Denmark and via networks of networks. Two LGBTQ+ young adults living with chronic conditions held the dialogues. Mode of communication was chosen by the participants (either face-to-face, via internet or via telephone). Data analysis was conducted via the “analysis grid” (Roest et al. 2021).

**Results:**

Nine dialogues lasting from 20 to 50 minutes were held with youth from 14 to 24 years old during spring 2022. According to their narratives, supportive and safe learning environments would: respect for change of names and pronouns, update learning materials, allow for flipped classrooms (hybrid teaching tested under COVID lockdowns), have separate neutral change rooms/bathrooms and create safe private spaces to take medications. They would also permit higher absenteeism rates for those living with chronic conditions or getting hormonal treatments, allow for more breaks/slower version of the pensum, and show proactive healthy curiosity and respect for “invisible diseases”, fluid SOGIEs and neurodiversity/neurodivergent profiles.

**Conclusions:**

The differing participants’ narratives provide innovative ways to create safe and supportive inclusive learning environment that embrace and enhance diversity.

